# Dynamics of Inflammatory Response in Autoinflammatory Disorders: Autonomous and Hyperinflammatory States

**DOI:** 10.3389/fimmu.2018.02422

**Published:** 2018-10-17

**Authors:** Ahmet Gül

**Affiliations:** Division of Rheumatology, Department of Internal Medicine, Istanbul Faculty of Medicine, Istanbul University, Istanbul, Turkey

**Keywords:** inflammation, autoimmunity, autoinflammatory disorders, innate immunity, hyperinflammatory response, autonomous inflammation, trained immunity, innate tolerance

## Abstract

Autoinflammatory diseases were originally defined as a group of monogenic disorders associated with seemingly unprovoked inflammatory episodes mediated mainly by the innate immune system and without direct involvement of adaptive immunity. The renewed concept encompasses a larger group of disorders including multifactorial diseases, which share the same inflammatory and clinical features with the monogenic disorders. Coining of the “auto” prefix to these inflammatory diseases suggests a constitutively active and self-augmenting innate immune response, but only a subgroup of them including cryopyrin-associated periodic syndrome (CAPS), associated with dominantly inherited gain-of-function *NLRP3* variants, fits well with the definition of the “autonomous” inflammatory conditions. However, the “autoinflammation” concept also includes another group of disorders characterized by episodes of exaggerated inflammatory response only when challenged by certain triggers. The dynamics of this latter group can be better defined as a “hyperinflammatory” state, which shares similar characteristics with the innate memory or trained immunity. Differentiation of “autonomous” and “hyperinflammatory” states of autoinflammatory disorders can provide additional insights to understand their pathogenesis and develop better management strategies since both conditions may have different inflammatory dynamics affecting the severity and frequency of clinical findings and treatment responses.

“Heat not a furnace for your foe so hot

That it do singe yourself.”

Henry VIII

William Shakespeare

## Introduction

Inflammation is a physiologic process aiming to protect the integrity of organisms against exogenous or endogenous dangerous insults (1–3). This process involves the recognition of pathogen—or danger associated molecular patterns by relevant receptors, which leads to the development of a response involving different cells and mediators to eliminate or limit the threat; and this response resolves with the repair of damages to restore the homeostasis.

The inflammatory response is well regulated to ensure a controlled reaction limited only to the pathogens or dangerous insults. During the turn of the twentieth century, Paul Ehrlich used the “*horror autotoxicus*” term to describe organisms' ability to recognize the self and not develop a harmful response against self-tissues (4). Although the regulatory mechanisms involved in inflammatory response work perfectly well in general, the problems in these mechanisms are considered to be responsible for the development of so-called “inflammatory” disorders (5). Autoimmune diseases are the most widely known examples of the “*autotoxic*” inflammatory disorders, and they do develop as a result of failures in the immune tolerance mechanisms causing the persistent activity of pathogenic self-reactive T and B cells.

On the other hand, regulatory problems in the innate immune response have been identified as the underlying pathology of another subset of inflammatory disorders, and uncontrolled or disproportionate innate inflammatory response triggered by the recognition of pathogen—or danger-associated molecular patterns has been shown to be responsible for the clinical and pathologic findings (6). Hereditary periodic fever syndromes constitute the best examples of this subgroup, and these conditions have been named as “*autoinflammatory disorders*” to differentiate them from “*autoimmune diseases*”, following the identification of genetic basis of familial Mediterranean fever (FMF) and tumor necrosis factor receptor associated periodic syndrome (TRAPS). Autoinflammatory disorders have originally been described as pathological conditions associated with seemingly unprovoked episodes of inflammatory response mainly involving the innate immune system with excessive production of proinflammatory cytokines and chemokines and without direct role of pathogenic autoantibodies or cellular immunity against self-antigens (6–8). An updated definition of this group encompasses a larger spectrum of disorders including multifactorial diseases, which share the same inflammatory characteristics and clinical features with the monogenic disorders (8, 9).

Following the identification of several new members of the autoinflammatory disorders, different approaches have been used to classify them. Classification attempts have mainly been based on the mechanisms affecting the regulation of innate immune response or the types of over-produced cytokines/inflammatory mediators involved in their pathogenesis (6, 8). Regulatory defects may rely on the constitutive activation of intracellular pathogen—or danger-associated signal sensing, intracellular accumulation of signals triggering innate sensors, loss of the negative regulatory function of innate response proteins, or up-regulation of post-receptor signaling mechanisms in innate immunity (8). Depending on the dysregulated pathways, inborn errors result in increased production of particular cytokines, such as interleukin 1 beta (IL-1β) or type 1 interferon, or up-regulated secretion of several proinflammatory cytokines and chemokines in a more complex way (6, 8).

This review aims to discuss the dynamics of inflammatory response during the course of the autoinflammatory disorders within the context of trained immunity.

## Autonomous vs. hyperinflammatory states in autoinflammatory disorders

In monogenic autoinflammatory disorders, increased inflammatory response develops as a result of gain-of-function or loss-of-function mutations in the genes involved in the innate immune response (8). Cryopyrin-associated periodic syndromes (CAPS) typically represent the autoinflammatory mechanisms associated with gain-of-function mutations in the *NLRP3* gene, which result in increased constitutive activity of the intracellular sensor protein (10). Mutation-dependent conformational changes in the NLRP3 protein result in increased production of IL-1β and a clinical spectrum ranging from the self-limited inflammatory episodes to the persistent severe inflammation (10, 11). In the mildest end of the spectrum, CAPS patients develop a “hyperinflammatory” response only when they are exposed to cold (Table 1). However, in the severe end, which was previously called as *Neonatal Onset Multisystem Inflammatory Disorder* (NOMID), patients start to have an “autonomously” increased IL-1β production starting within the first year of life (Table 1). Somatic mosaicism in myeloid cell lineages for the *NLRP3* gene mutations may be enough for the development of disease manifestations associated with moderate to severe inflammation, and expansion of the mutated clone with the passage of time may be the cause of the late onset of clinical findings in some patients (12–17).

**Table 1:** Possible contributions of autonomous and hyperinflammatory states to the clinical findings of common monogenic autoinflammatory disorders with putative scores based on the characteristics of clinical findings.

**Disease**	**Gene**	**Hyperinflammatory state**	**Autonomous inflammatory state**
CAPS (FCAS)	*NLRP3*	++	+
CAPS (NOMID)	*NLRP3*	++	++++
FMF	*MEFV*	+++	+
crFMF	*MEFV*	+	+++
PAAND	*MEFV*	+	++++
Blau syndrome	*NOD2*	+	+++
Crohn disease	*NOD2*	+++	+
MKD	*MVK*	++	+++
TRAPS	*TNFRSF1A*	+++	++

*Abbreviations. CAPS, Cryopyrin associated periodic syndrome; FCAS, Familial cold autoinflammatory syndrome; NOMID, Neonatal-onset multisystem inflammatory disease; FMF, Familial Mediterranean fever; crFMF, colchicine refractory-familial Mediterranean fever; PAAND, Pyrin-associated autoinflammation with neutrophilic dermatosis; MKD, Mevalonate kinase deficiency; TRAPS, TNF receptor associated periodic syndrome*.

On the other hand, Familial Mediterranean Fever (FMF), the most common form of the autoinflammatory disorders, is associated with autosomal recessively inherited variants in exon 10 of the *MEFV* gene, which encodes the pyrin protein. Pyrin has been linked to different roles in the regulation of the inflammasome complex, and monocytes of FMF patients produce increased amount of IL-1β depending on the number of penetrant exon 10 mutations, only when stimulated with proinflammatory environmental triggers such as lipopolysaccharide (18). The variants associated with increased IL-1β production do not interfere with the production of regulatory natural antagonist protein IL-1Ra, which help limit the inflammatory episode within 2–3 days (18). Most of the FMF patients do not have constitutively enhanced autonomous production of IL-1β, and it is usually not possible to detect ongoing inflammation in between these “hyperinflammatory” episodes.

However, patients carrying dominantly inherited p.Ser242Arg mutation in exon 2 of the *MEFV* gene develop a different inflammatory phenotype, which is related to the constitutively active pyrin-inflammasome and continuously elevated IL-1β-driven acute phase response (19). The clinical picture associated with this *MEFV* variant has been named as pyrin-associated autoinflammation with neutrophilic dermatosis (PAAND); and it is characterized by fever lasting several weeks rather than days, neutrophilic dermatosis, arthralgia, myalgia, cardiomyopathy, anemia, pyogenic arthritis, and serositis (19). Phosphorylation of serine at position 242 of the pyrin protein is critical for the binding of its negative regulator 14-3-3 protein; and this regulatory mechanism is linked to the guard function of pyrin protein as a sensor of RhoA-mediated changes within the cytoplasm. Various bacterial toxins affecting the functions of Rho GTPases, such as TcdB toxin of *Clostridium difficile* and ADP-ribosylating C3 toxin of *Clostridium botulinum*, can induce pyrin-inflammasome through the decreased downstream activity of RhoA protein, which affects the binding of 14-3-3 to pyrin (19–21). Missense changes in the one of the phosphorylation sites of pyrin can mimic the intracellular changes induced by these bacterial toxins and result in autonomous activation of pyrin-inflammasome with more persistent inflammatory dynamics different from the characteristics of FMF.

Similarly, variants in the *NOD2 (CARD15)* gene are linked to both autonomous and hyperinflammatory disorders. Dominantly inherited gain-of-function mutations in the NACHT domain of the *NOD2* gene lead to the increased basal activity of NF-κB. This autonomous inflammatory response is associated with Blau syndrome, which is characterized by early-onset granulomatous uveitis, dermatitis, and arthritis with camptodactily deformities (22, 23). On the other hand, loss-of-function variants in the leucine-rich repeat (LRR) region of the *NOD2* gene are associated with the multifactorial Crohn disease; and these mutations are thought to be associated with dysregulated interaction between host and dysbiotic intestinal microbiota leading to hyperinflammatory responses (23–26).

To prevent the confusion associated with the “hyperinflammatory” state, it is necessary to note that a group of heterogeneous disorders have been grouped under the term of “hyperinflammatory syndromes,” because of a common immunopathology associated with a cytokine storm or hypercytokinemia; which includes one of the hereditary autoinflammatory disorders, familial hemophagocytic lymphohistiocytosis (27). The hyperinflammatory response constitutes the shared pathogenic mechanism between hemophagocytic lymphohistiocytosis and macrophage activation syndromes, and the latter condition can develop in various autoinflammatory and autoimmune settings ranging from systemic onset juvenile idiopathic arthritis, Kawasaki disease to systemic lupus erythematosus (27). The hyperinflammatory syndromes associated with dysregulated cytokine production or cytotoxicity defects can also be seen in association with infections, malignancies, and immunodeficiency syndromes such as Chédiak Higashi, Griscelli 2, Hermansky Pudlak 2 syndromes (27). Infections are usually considered as the main triggers of hyperinflammation, which may be an example of maladaptive “trained immunity” response.

## Trained immunity and innate tolerance

**Figure 1:**
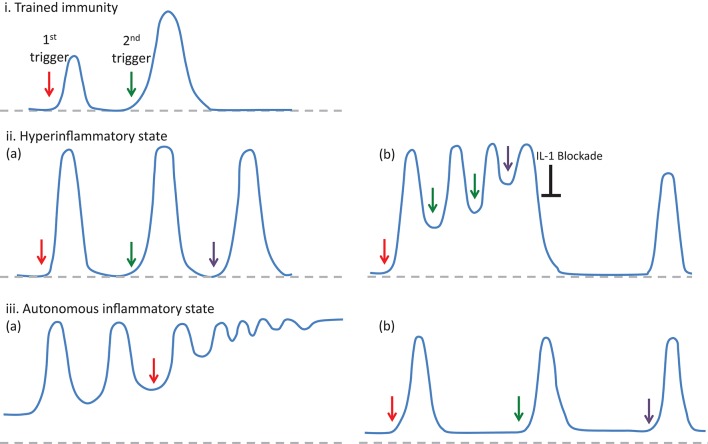
The features of inflammatory responses developing in the trained immunity (i) and autoinflammatory disorders (ii and iii), in regard to the intensity and frequency of episodes and their resolution to the baseline. In trained immunity (i), after the resolution of the inflammatory response following an infection or vaccination (1st trigger), a following stimulation with different pathogens (2nd trigger) results in a stronger inflammatory response (28). In autoinflammatory disorders, triggering factors (arrows) may either induce a hyperinflammatory state (ii-a), defined as an enhanced inflammatory response developing after a stimulation, or an autonomous inflammatory state (iii-a) associated with gain of function mutations, which lead to the continuous production of IL-1β and ongoing inflammatory activity in between attacks. However, in autoinflammatory disorders associated with the hyperinflammatory dynamics, some patients may experience “autonomous” inflammatory states (ii-b), which require a therapeutic intervention to reset the inflammatory dynamics. On the other hand, in some autoinflammatory disorders associated with autonomous inflammatory characteristics, disease course may be very mild and obvious inflammatory findings could only be detected when the patients are exposed to triggering factors such as cold (iii-b).

It has long been suggested that one of the critical differences between adaptive and innate immunity is that adaptive immune response can build immunological memory but innate immunity cannot (28). However, several recent studies have demonstrated that an innate version of immunological memory can be induced after infections or vaccinations, which results in a stronger inflammatory response with broader specificity following a secondary stimulation with different pathogens (Figures 1i) (28–30). This weeks or months-lasting memory is named as “trained immunity,” and it is mainly associated with epigenetic re-programming of innate immune cells, especially of the cells of myeloid lineage (30). This stronger inflammatory response to various microbial triggers following the initial infections or vaccinations involves both histone modifications (i.e., H3K4me1, H3K4me3, H2K27Ac, H3K9me2), and metabolic changes (i.e., increased aerobic glycolysis through the mTOR pathway and increased production of mevalonate) in those cells (30, 31).

However, maladaptive conditions associated with inappropriate activation of trained immunity may result in immunodeficiency states or hyperinflammatory responses (28, 32). Inappropriate exposure of innate immune cells to bacterial endotoxins such as lipopolysaccharide (LPS) may result in a refractory state to subsequent challenges of LPS, which is known as “endotoxin tolerance,” and it contributes to the immune paralysis observed in patients with sepsis (33, 34). Although several findings suggest changes in the polarization and cytokine production pattern of inflammatory cells, exact mechanism of the endotoxin tolerance has yet to be clarified (33, 35).

In the other end of the maladaptive conditions, hyperinflammatory responses due to induction of trained immunity may contribute to the pathogenesis and course of monogenic autoinflammatory disorders as well as several other inflammatory conditions (32), which may show variability in the expression and severity depending on the environmental factors (36, 37).

## Trained immunity and autoinflammatory disorders

Non-specific hyperinflammatory response to a broad spectrum of triggers was first described as the “pathergy” reaction in 1933 by Rössle, and his definition corresponds well with the current understanding of the trained immunity associated with sustained changes in the expression of proinflammatory cytokines due to epigenetic modifications (38, 39). Therefore, long-standing observations on the pathergic response and trained immunity regarding the intensity of inflammatory response to various triggers may also have a potential to analyze the variability of clinical findings in the course of the autoinflammatory disorders, which cannot be explained by only genotype (32).

Investigation of pathogenic mechanisms associated with autosomal recessively inherited Mevalonate Kinase Deficiency (MKD) may provide the most direct clues for the role of trained immunity in autoinflammatory disorders. Recently, it has been shown that activation of the cholesterol synthesis pathway, but not the synthesis of cholesterol molecule itself, is involved in the stimulation of trained immunity, and mevalonate is the critical molecule of this pathway in the induction of epigenetic changes, such as the H3K4me3 change at the promoter regions of proinflammatory *TNFA* and *IL6* genes (31).

Retention of intracellular mevalonate in the monocytes of MKD patients due to decreased mevalonate kinase enzyme activity has also been shown to be associated with the same trained immunity phenotype, which leads to the autoinflammatory response (31). Prenylation defects associated mevalonate kinase enzyme defects has also been linked to the decreased RhoA-associated phosphorylation of pyrin protein and activation of the pyrin inflammasome (21). In addition to the mutation-specific conformational changes affecting the mevalonate kinase enzyme activity, the extent of epigenetic changes are expected to contribute to the variability in the clinical spectrum, ranging from the hyperinflammatory response due to temporary increases in the mevalonate concentration following stimuli such as vaccination in the mild end to the autonomous hyperinflammatory response resulting from constitutively increased production of mevalonate due to severely defective activity of the enzyme leading to the sustained changes (8, 40).

Similar to the *MVK* gene variants leading to MKD, the rare genetic variants responsible for other hereditary autoinflammatory disorders can also be assumed to be associated with a much stronger and durable “trained immunity” response leading to maladaptive conditions (Figure 1). For example, country-dependent environmental factors affecting the risk of infections and infant mortality rate show an association with the risk of amyloidosis in FMF patients (37). In addition to the rare variants, even some common polymorphisms associated with multifactorial autoinflammatory disorders such as the NOD2 variants in Crohn disease may be involved in “hyperreactive” innate response and maladaptive trained immunity following an infection or associated with dysbiotic microbiota.

It may also be helpful to note that in the autoimmunity end of the inflammatory disease spectrum, more than half of the disease associated non-coding variants have been mapped to enhancer-like elements in immune cells, especially lymphocytes, possibly altering non-canonical regulatory sequences and causing context-dependent autoimmune responses (41).

## Implications of defining hyper-versus autonomous inflammatory states

Identification of molecular basis of several autoinflammatory disorders has led to development of targeted treatments with successful results such as IL-1 blocking agents in inflammasomopathies; and their classification according to underlying inflammatory pathways proved to be useful in explaining both the pathogenesis of clinical findings and variability in the treatment responses. On the other hand, addition of another dimension to the classification, by considering the dynamics of inflammatory response associated with hyperreactive or autonomously active innate immune cells may provide further benefits in the interpretation of clinical findings and developing better management strategies with the optimum use of available treatment options in individual patients (Table 1).

Bozkurt et al. developed a unifying mathematical model to understand the dynamics of recurrent nature of inflammation in FMF and CAPS, in the form of coupled nonlinear ordinary differential equations (42). Comprehensive bifurcation analyses of the model revealed that the concentration of active caspase 1 enzyme is the most critical parameter determining the healthy state as well as the inflammatory features of FMF and CAPS patients (42). In FMF patients, a self-limited inflammatory episode develops only when the system is triggered by an insult, compatible with the “hyperinflammatory” state (Figures 1ii-a). On the other hand, gain-of-function mutations in CAPS patients result in constitutively active caspase 1 leading to an autonomous periodicity with episodes developing even when there is no trigger (Figures 1iii-a) (42). In patients with low-penetrance variants periodicity of the attacks may decrease, but when the variants are penetrant and triggers are present, patients may develop a non-oscillatory, continuous inflammation representing the most severe end of the CAPS spectrum, NOMID (Table 1) (42).

In clinical practice, the inflammatory characteristics of a subgroup of FMF patients with inadequate response to colchicine treatment (also named as colchicine refractory-FMF patients) can be classified as an “autonomous” state due to genetic and/or environmental factors affecting the duration and sustainability of caspase 1 activity (Table 1). In some of the FMF patients, this autonomous inflammatory state may be temporary due to intervening stressful conditions resulting in a vicious circle characterized by continuous production of pro-inflammatory cytokines, which causes either unexpectedly long episodes or very frequently recurring attacks despite highest tolerable doses of colchicine along with an elevated acute phase response in between attacks (43). In this setting, blocking the activity of IL-1 by biologic agents may reset the autonomous production of IL-1β, and some of these colchicine refractory-FMF patients may experience a stable disease course with regained good response to colchicine (Figures 1ii-b) (44).

Within the same context, some patients with an autoinflammatory disorder characterized by gain-of-function mutations and associated with autonomous production of IL-1 may run a milder disease course with very rare inflammatory episodes (Table 1). In this situation, despite autonomous production of IL-1 at low level, inflammatory clinical findings can only be triggered following strong stimuli such as cold exposure, infections, or vaccinations, and they may not need continuous blockade of IL-1 to control inflammatory episodes (Figures 1iii–b).

Similarly, inhibition of IL-1 activity with potent drugs may reset the IL-1β-dependent vicious circle and cytokine-driven pathologies in patients with higher constitutive caspase 1 activity; and following a single high-dose administration of anti-IL-1β monoclonal antibody, some CAPS patients may not require additional treatment for months due to inhibition of IL-1β-dependent production of IL-1, which may be increased up-to-5-fold compared to healthy controls (10). Also, DNA methylation status of CAPS patients may become similar to that of healthy controls when they are using anti-IL-1 treatments, which suggests sustained improvements in the epigenetic programming (45).

## Conclusions

In conclusion, adding the “hyper” or “autonomous” as well as the trained immunity dimensions to the concept of autoinflammation could provide further benefits for both understanding of the immunopathogenesis of the variable disease course in these conditions and developing better strategies for the management of inflammatory findings in regard to the timing, dosage, and administration intervals of IL-1 blocking agents.

## Author contributions

The author confirms being the sole contributor of this work and has approved it for publication.

### Conflict of interest statement

AG declares that he received consulting fees and study support from Novartis, Servier, and TR-Pharm.
